# The Quantitative Genetic Control of Root Architecture in Maize

**DOI:** 10.1093/pcp/pcy141

**Published:** 2018-07-18

**Authors:** Adam L Bray, Christopher N Topp

**Affiliations:** 1 Division of Plant Sciences, University of Missouri-Columbia, Columbia, MO, USA; 2 Donald Danforth Plant Science Center, St. Louis, MO, USA

**Keywords:** Genotype to phenotype, Imaging and analysis, Quantitative genetics, Root architecture, Root mutant, *Zea mays*

## Abstract

Roots remain an underexplored frontier in plant genetics despite their well-known influence on plant development, agricultural performance and competition in the wild. Visualizing and measuring root structures and their growth is vastly more difficult than characterizing aboveground parts of the plant and is often simply avoided. The majority of research on maize root systems has focused on their anatomy, physiology, development and soil interaction, but much less is known about the genetics that control quantitative traits. In maize, seven root development genes have been cloned using mutagenesis, but no genes underlying the many root-related quantitative trait loci (QTLs) have been identified. In this review, we discuss whether the maize mutants known to control root development may also influence quantitative aspects of root architecture, including the extent to which they overlap with the most recent maize root trait QTLs. We highlight specific challenges and anticipate the impacts that emerging technologies, especially computational approaches, may have toward the identification of genes controlling root quantitative traits.

## Introduction

Plant physiologists have spent many years thoughtfully characterizing roots in fine detail, and creating an intricate vocabulary to describe their observations. Common terminologies have been proposed at various times by the International Society of Root Research (ISRR), the Plant Ontology Consortium and maize researchers (Feix et�al. 2002, Ilic et�al. 2007, Hochholdinger 2009, Zobel and Waisel 2010). Root traits have generally been grouped based on developmental criteria (tissue of origin), anatomy (internal structure at the tissue or cellular level) and topology (branching order or connectedness, i.e. primary, secondary, tertiary, etc.). These groupings have been and continue to be broadly used; however, as technologies advance, new ways to characterize roots arise.

Root system architecture (RSA) has been defined as the growth pattern of roots within, and in response to, their environment, i.e. where, when and what type of roots grow (Fitter 1987). RSA integrates aspects of development and topology, and more recently has been connected to anatomical features (Burton et�al. 2013, Lynch et�al. 2014). Root architecture is measured as a function of increasing root system complexity through plant development, which has been facilitated by technologies that quantify many traits with high throughput. This increased scope has allowed plant geneticists to begin identifying the genetic basis of root traits across a wide spectrum of descriptors.

*Zea mays* (L.) is one of the most highly produced grain crops in the world and has been the focus of extensive breeding and genetic research over the years. However, maize roots have received far less attention compared with aboveground structures because they are hidden from view and grow in complex patterns that are heavily influenced by the soil environment. Indirect selection for root architecture in response to denser planting is posited as a major factor for increased yield over the past half century in the USA (Hammer et al. 2009), but no genes have been identified or attributed to this phenomenon.

As currently understood, developmentally defined maize root types are controlled by single genes that regulate gross phenotypic differences, often presence or absence (Hochholdinger et�al. 2018). Root architecture and anatomical traits appear instead to be quantitative and controlled by many genes (Price and Tomos 1997, Mano et�al. 2007, Topp et�al. 2013, Burton et�al. 2015, Zurek et�al. 2015). These traits also have a well-documented capacity for phenotypic plasticity (MacMillan et�al. 2006, Rosas et�al. 2013, Meij�n et�al. 2014) that can contribute to crop yield stability across multiple environments (Sandhu et�al. 2016). Quantitative variation for root architecture has been proposed to facilitate trait optimization in different environments by concerted ‘fine-tuning’ of many loci (Gifford et�al. 2013, Rosas et�al. 2013). However, this idea has not been well tested in crop plants, largely because few genes have been identified that control root architecture. Thus, fully realizing the value of root architecture for crop improvement (Lynch 1995, 2013, de Dorlodot et�al. 2007, Hochholdinger and Tuberosa 2009) will require a more thorough understanding of the specific genetic loci involved in quantitative root variation.

Only a small number of genes that control strong developmental phenotypes have been cloned in maize (Hochholdinger et�al. 2018). Additionally, many root quantitative trait locus (QTL) mapping studies and meta-analyses spanning decades of work have been conducted (Hund et�al. 2011), but no causal genes have been reported yet. Amid these data, important questions remain. (i) To what extent could the known major genetic regulators also control quantitative variation, and do they co-align with maize root QTLs? (ii) What are the key experimental factors limiting our current ability to identify the genetic basis of quantitative root traits? (iii) What tools can be leveraged to help survey the diversity of root traits in maize going forward?

## Is There a Distinction Between Qualitative and Quantitative Regulators of Root Architecture?

Maize root system development can first be divided into an embryonic root system derived from the seed and the post-embryonic root system derived from the shoot, which eventually dominates the root architecture. The embryonic root system is made up of a primary root and a variable number of seminal roots depending on genotype and environment (Kiesselbach 1949, Sass 1977, Feldman 1994). The post-embryonic root system is comprised of lateral roots and whorls of nodal roots, which are called crown roots if below the soil and brace or prop roots if above the soil. Nodal roots emerge at the base of each node, and so comprise a temporal and developmental axis that increases shootward. Lateral roots initiate from the pericycle of both embryonic and post-embryonic roots, and comprise the first and all subsequent topological orders of branching (Bodner et�al. 2013). Root hairs are single-celled protrusions that initiate from the epidermis of all root types and have been considered both anatomical and developmental structures. The characterization of mutant phenotypes in this way has led to the identification of 12 maize developmental root mutants, which are summarized in Table�1.

**Table 1:** Summary of known maize root development mutants and cloned genes

Root type	Mutagen	Genetic background	Introgressed	Mutant phenotype	Gene name		GeneID^a^	Bin	Reference
Root hair	Mu	Robertson mutator stocks	Q60, Aet/LineC, BMS, NY821, B37	Root hairs do not elongate	*rth1*	*roothairless 1*	GRMZM2G099056	1.09	Wen et al. (2005)
			NA	Root hairs 1/4 length of wild type	*rth2*	*roothairless 2*	NA	5.04	Wen and Schnable (1994)
			NA	Root hairs do not elongate; ‘stocking cap’ phenotype	*rth3*	*roothairless 3*	GRMZM2G377215	1.01	Hochholdinger et al. (2008)
	EMS	Pioneer inbred	B73, Mo17	Root hair initiation and elongation	*rth5*	*roothairless 5*	GRMZM2G426953	3.06	Nestler et al. (2014)
	Mu, AcDs	Pioneer inbred, W22	B73	Root hairs are arrested after bulge formation	*rth6*	*roothairless 6*	GRMZM2G436299	1.05	Li et al. (2016)
Embryonic lateral	EMS	B73	A632	Lateral root primordia not present on embryonic roots	*lrt1*	*lateral rootless 1*	NA	2S	Hochholdinger and Feix (1998)
Lateral	Mu	SWS2000	A632, B73	Lateral roots 1/4 length of wild type	*slr1*	*short lateral roots 1*	NA	NA	Hochholdinger et al. (2001)
		NA	NA	Lateral roots 1/4 length of wild type	*slr2*	*short lateral roots 2*	NA	NA	
Lateral and seminal	Mu	B73	F2, F7, Aet, HiIIA	Seminal and lateral root primordia absent on embryonic roots; delayed gravitropic response	*rum1*	*rootless with undetectable meristem 1*	GRMZM2G037368	3.08	von Behrens et al. (2011), Woll et al. (2005)
Seminal and nodal	Enhancer, Mu, AcDs	DK105	A632, B73, W22, W23	No nodal or seminal roots	*rtcs*	*rootless concerning seminal and crown roots*	GRMZM2G092542	1.01	Taramino et al. (2007)
Nodal	Mu	Pioneer inbred	NA	Nodal root length reduced	*rtcl*	*rtcs-like*	AC149818.2_FGT009	9.07	Taramino et al. (2007), Xu et al. (2015)
Nodal	Unknown	NA	NA	Lateral roots few or absent, reduced crown root number, no aerial brace roots	*rt1*	*rootless 1*	NA	3S	Jenkins (1930)
No mutant phenotype			NA	Homolog of *rum1*^b^	*Rul1*	*Rum1-like*	GRMZM2G163848	8.06	von Behrens et al. (2011), Zhang et al. (2016)
			NA	Blast of *AtLRP1*; regulated by *rum1*^b^	*Lrp1*	*Lateral root primordia 1*	GRMZM2G077752	8.06	Zhang et al. (2015)

^a^GeneID is from B73 v3 reference.

^b^These genes were identified by homology to *rum1* and *AtLRP1*, and are predicted to have similar phenotypes but no mutants exist to validate these phenotypes.

Forward genetics approaches have been used to generate mutant alleles of genes involved in maize root anatomy and development. Of the 12 root mutants: two affect seminal root development, three affect shoot-borne root development, four affect lateral root development and five affect root hair development. Only seven of these genes have been cloned (Table�1), and they have been thoroughly reviewed elsewhere (Hochholdinger 2009, Hochholdinger and Tuberosa 2009, Yu et�al. 2016, Hochholdinger et�al. 2018). Mutants have traditionally been screened by eye at the seedling stage (10–14 d after germination) from plants grown in paper towel rolls (Hochholdinger 2009). This screening method allows for rapid scoring of many lines, but is biased toward identifying obvious root phenotypes that affect qualitative aspects of external morphology rather than subtle variation of quantitative phenotypes. Not surprisingly, the genes controlling the most obvious and severe mutants were cloned first. For example, the first cloned mutant was *rth1* which has no root hairs visible by eye on the emerging primary root at 3 d after germination (Wen et�al. 2005; Andorf et�al. 2016; images at https://maizegdb.org/data_center/variation?id=61056 (June 29, 2018, date last accessed)). The mutants *rth2*, *slr1* and *slr2* have more subtle phenotypes and have not been cloned. Root hairs of the *rth2* mutant elongate but are shorter than those of the wild type (Wen and Schnable 1994). Lateral root length of both *slr1* and *slr2* mutants is reduced compared with the wild type (Hochholdinger et�al. 2001). The mutant *lrt1* has a strong effect on the embryonic root system, which has no laterals (Hochholdinger and Feix 1998). However, the nodal root system has apparently normal lateral roots yet is smaller than the wild type, raising the possibility that the *lrt1* phenotype may be more quantitative when evaluated at a later growth stage. More robust phenotyping of root mutants, especially throughout development, may aid in gene identification and reveal more quantitative aspects of their phenotype.

Due to the enormous amount of structural, genetic and transcriptional variation known in the maize pan-genome, any mutagenesis study is limited to the variation present in the targeted genotypes (Hirsch et�al. 2014, Jiao et�al. 2017). For example, the ‘B73’ genome only captures an estimated 70% of non-transposon genes present in diverse maize inbreds (Gore et�al. 2009). Additionally, approximately 30% of genes in both ‘B73’ and ‘PH207’ show genotype-specific expression (Hirsch et�al. 2016), suggesting that the genomic environment could also have a significant effect on the phenotypic expression of a mutant allele. While not extensively studied, epistatic modifiers have been observed for half of the mutants when they were crossed or mutated in multiple genetic backgrounds: *rum1*, *rtcs*, *slr1* and *rth2* (Table�1). The epistatic modifiers of *rtcs* and *rum1* are paralogs, known as *Rtcl* and *Rul1*, respectively (Taramino et�al. 2007, von Behrens et�al. 2011, Xu et�al. 2015, Zhang et�al. 2016). No mutants have been identified for *Rul1* but, interestingly, the *rtcl* mutant has a less severe phenotype than *rtcs*, resulting in normal seminal roots and reduced length of crown roots. This observation supports the finding from Schnable and Freeling (2011) that genes from the maize1 subgenome are over-represented in the historic identification of visible mutant phenotypes because of their increased severity. Given that the ancestral duplication of the maize genome led to widespread gene differentiation (Schnable et�al. 2011), it is tantalizing to wonder if the maize2 subgenome paralogs of root developmental genes may generally be more quantitative in nature, and thus more likely to control root system architecture tunability. It will be important to screen diverse maize lines—including inbreds, landrace, and wild teosinte ancestors—if we are to obtain a complete picture of the phenotypic context of a given mutation and any potentially adaptive alleles (Schmidt et�al. 2016, Topp et�al. 2016).

To date, only eight of the 12 known root mutants have been introgressed into at least two genetic backgrounds (Table�1). There has been little further work on quantitative aspects of these phenotypes, with one notable exception: Abdel-Ghani et�al. (2015) directly compared quantitative phenotypic variation across different maize lines to identify associations with functional alleles of root development genes. They queried an association panel of 74 maize inbreds, and reported that allele variants for *Rtcl*, *Rth3*, *Rum1* and *Rul1* contributed to quantitative variation of seedling root traits. However, the lack of genome-wide markers in this experiment made it difficult to understand how these targeted developmental gene associations compare with genome-wide associations with root traits. When Pace et�al. (2015) conducted a genome-wide association study of a different but overlapping set of inbred lines for seedling RSA traits, *Rum1* was the only known root gene with significant marker associations nearby. Several other maize root QTLs overlap with or have been found in proximity to known root mutant genes, yet no causal relationship has been identified between genes and the large QTL regions. QTLs for seminal root number, crown root length, brace root whorl number and crown root number overlap with *Rul1* (Pestsova et�al. 2016, Salvi et�al. 2016, Gu et�al. 2017, Liu et�al. 2017, Zhang et�al. 2018). *Rum1* was under QTLs for seminal root number, lateral root density, lateral root length and crown root number per whorl (Pestsova et�al. 2016, Salvi et�al. 2016, Liu et�al. 2017, Zhang et�al. 2018). *Rtcl* was under QTLs for lateral root density and brace root number (Pestsova et�al. 2016, Zhang et�al. 2018), and *Rtcs* was found under a QTL for seminal root number (Salvi et�al. 2016). In total, the evidence suggests that known root mutant genes may plausibly control quantitative variation for maize RSA; however, as most QTL regions contain hundreds of genes, more research is needed to identify bona fide causal relationships.

## Current Knowledge and Challenges Towards Gene Identification of Maize Root Architecture QTLs

While the list of QTLs controlling maize root system traits is long, no causative genes underlying any of these QTLs have been reported. In Table�2 we summarize the 19 maize root QTL mapping and genome-wide association studies (GWAS) that have been published since or were not included in the last summary (Hund et�al. 2011). These works were based on 12 biparental populations (19 inbreds and 2 teosintes) and three association populations (396 and 384 inbreds, and 66 landrace F_1_ doubled haploids).

**Table 2:** Summary of 19 maize root trait QTLs and GWAS studies that have been published since or were not included in Hund et al. (2011)

Trait group	Trait measurement	No. of traits	Population	Pop size	Rep� Repeat	Plants per rep	Time and/ or stage sampled	No. of markers	Map density	Media and treatment	*H*^2^	No. of QTLs	Meta- QTLs^a^	Reference
Nodal root anatomy	Aerenchyma: visual score	1	B64�*Z. nicaraguensis*: F_2_	141	1 � 1	1	28 d V6	85 SSRs	17.2 cM	Granular soil; GH	NA	6	N	Mano et al. (2007)
			*Z. nicaraguensis*�Mi29: BC_2_F_1_	214	1 � 1	1	28 d V6	94 SSRs	10.2 cM	Granular soil; GH	NA	3	N	Mano and Omori (2008)
			*Z. nicaraguensis*�Mi29: BC_4_F_1_	123	1 � 1	1	28 d V6	156 SSRs + 38 Indels	NA	Granular soil; GH	NA	2	N	Mano and Omori (2009)
	ROL: methylene blue + oxygen electrode	2	*Z. nicaraguensis*�Mi29: BC_3_F_5_	48	4 � 1	1	25 d	98 SSRs	NA	Hydroponics	NA	1	N	Watanabe et al. (2017)
	RootScan: cross-sections	10	B73�Mo17 (IBM): RILs	200	3 � 1	1;3	30 d V6; 56 d V12	8,224 GBS-SNPs	0.7 cM	Potting mix; field	0.47 to 0.78	1	N	Burton et al. (2015)
			Oh43�W64a (OhW): RILs	200	3 � 1	1	30 d V6	5,683 GBS-SNPs	0.6 cM	Potting mix	0.47 to 0.78	5	N	
			NY821�H99 (NyH): RILs	176	3 � 1	1	30 d V6	5,320 GBS-SNPs	0.7 cM	Potting mix	0.47 to 0.78	0	N	
Nodal root	Visual count	2	Huangzao 4� CML288: IF_2_	278	1 x 1	10	VT + 10 d	237 SSRs	12.3 cM	Field	NA	15	N	Ku et al. (2012)
			Huangzao 4�CML288: RILs	201	3 � 1	10	VT + 10 d	237 SSRs	8.33 cM	Field	NA	10	N	
	Visual count + weight	8	Yi17�Yi16: F_2_	276	1 � 1	1	R6	212 SSRs	7.35 cM	Field	0.42 to 0.79	44	Y	Gu et al. (2017)
			Yi17�Yi16: F2:3	241	2 � 3	8	R6	212 SSRs	7.35 cM	Field	0.40 to 0.66	49	Y	
	Visual count	9	*Z. parviglumis*�W22: BC_2_S_3_	866	1 � 4	10	VT per line	19,838 GBS-SNPs	39.6 kbp	Field	NA	133	N	Zhang et al. (2018)
RSA	WinRhizo + weight	7	NUEC2�NUEC4: DH	60	2 � 1	15-30	12 d	754 50k-SNPs	NA	Paper roll	NA	30	Y	Pestsova et al. (2016)
	Root count + weight	9	Ye478�Wu312: F_8_	218	3 � 3	2	20 d V5/6	184 SSRs	11.3cM	Hydroponics: HN + LN	0.36 to 0.69	134	Y	Li et al. (2015)
	Count + weight + WinRhizo	21	B73�Mo17 (IBM): RILs	200	3 � 1	1	30 d V6/7	8,224 GBS-SNPs	0.7 cM	Potting mix	0.10 to 0.60	7	N	Burton et al. (2014)
			Oh43�W64a (OhW): RILs	200	3 � 1	1	30 d V6/7	5,683 GBS-SNPs	0.6 cM	Potting mix	0.10 to 0.60	5	Y	
			NY821�H99 (NyH): RILs	176	3 � 1	1	30 d V6/7	5,320 GBS-SNPs	0.7 cM	Potting mix	0.10 to 0.60	3	Y	
	WinRhizo + weight	6	Ye478�Wu312: F_8_	218	3 � 2	2–3	50 d V6; 80 d VT; 120 d R6	184 SSRs	11.3 cM	Field	0.01 to 0.55	36	N	Cai et al. (2012)
			Ye478�Wu312: BC_4_F_3_	187	3 � 2	2–3	50 d V6; 80 d VT; 120 d R6	143 SSRs	11.3 cM	Field	0.01 to 0.55	36	N	
	WinRhizo + weight + length	8	Ye478�Wu312: F_8_	218	2;2;1	6; 12; 17	12 d; 8 d; 10 d	184 SSRs	11.3cM	Hydroponics; paper roll; vermiculite	0.52 to 0.83	46	Y	Liu et al. (2017)
			Ye478�Wu312: BC_4_F_3_	187	2;2;1	6; 12; 17	12 d; 8 d; 10 d	143 SSRs	11.3 cM		0.52 to 0.83	46	Y	
	Weight + volume	6	CIMMYT Asia Panel	396	2 � 2	1	VM	331,390 GBS-SNPs	6.2 kbp	Field in tubes	0.82 to 0.98	67	N	Zaidi et al. (2016)
	GiaRoots 3D	19	B73�Ki3 NAM RILs	175	3 � 1	1	4 d, 6 d, 8 d	1,106 SNPs	1.3 cM	Hoaglands gel	0.07 to 0.55	102	Y	Zurek et al. (2015)
	ARIA + weight	24	384 Ames Panel	384	3 � 1	3	14 d	135,311 GBS-SNPs	15.1 kbp	Paper roll	0.12 to 0.49	268	Y	Pace et al. (2015)
	Count + length + scans	10	B73�Gaspe Flint: BC_5_F_2_	75	2;2	9;5	7 d; 25 d	173 SSRs	NA	Paper roll; potting mix	0.69 to 0.95	14	N	Salvi et al. (2016)
	ARIA + weight	24	66 Landrace F_1_ DH	300	3 � 1	1	14d	62,077 GBS-SNPs	100 Mbp	Paper roll	0.06 to 0.50	39	N	Sanchez et al. (2018)
RPF	Dynamometer	1	Ye478�Wu312: F_8_	218	3 � 4	4	VT (silk) + 14 d	184 SSRs	11.3 cM	Field	0.61	4	Y	Liu et al. (2011)
			Ye478�Wu312: BC_4_F_3_	187	3 � 4	4	VT (silk) + 14 d	143 SSRs	11.3 cM	Field	0.44	3	Y	

RSA, root system architecture; RPF, root pulling force; ROL, radial oxygen loss; ARIA, automated root image analysis.

^a^QTL overlaps a meta-QTL: Y = yes, N = no.

Currently, the only genes reported to control a root architecture QTL in a crop species were found in rice: deeper rooting 1 (*DRO1*) (Uga et�al. 2013) and phosphorus starvation tolerance 1 (*PSTOL1*) (Gamuyao et�al. 2012). Both of these genes underlie stress tolerance traits and were identified in landrace germplasm rather than elite breeding lines, underscoring the importance of querying genetic diversity for root traits. A functional *DRO1* gene was found in a drought-tolerant cultivar ‘Kinandang Patong’ from the Philippines, whereas a 1 bp deletion caused a premature stop codon in the widely planted, drought-sensitive rice cultivar ‘IR64’. *PSTOL1* was found in the low phosphorus-tolerant aus-type ‘Kasalath’ in a 90 kb indel that is completely absent in the low phosphorus-intolerant ‘Nipponbare’ reference genome. The *DRO1* gene enhances root gravitropism, thus generating deeper roots, and PSTOL1 is a protein kinase involved in regulation of early crown root development that promotes root growth under both high and low phosphorus conditions. Both genes have been reported to enhance yield under their respective stress environments, with *DRO1* showing no yield penalty under well-watered conditions in some environments (Gamuyao et�al. 2012, Uga et�al. 2013). A study of *PSTOL1* orthologs in a sorghum diversity panel has shown a similar role for superior alleles of the gene in grain yield under low phosphorus conditions (Hufnagel et�al. 2014). *DRO1* and *PSTOL1* are powerful yet isolated examples of the potential for root architecture-based improvement of crops. Decades of work was needed for the identification of these genes in rice. Even though researchers have been studying maize root QTLs for over two decades (Lebreton et�al. 1995), similar success has not been achieved.

Gene identification is hampered by the combination of low root trait heritability and the size of mapping populations that can be adequately measured. Since phenotyping is laborious, practical considerations result in sparse or incomplete measurements of the root system. The strong environmental conditioning of root growth contributes to low heritabilities, especially in the field. Thus, few if any studies have had sufficient power to overcome the Beavis effect (Beavis 1994, Xu 2003), which states that the power to detect QTLs is directly proportional to the size of the mapping population and the heritability of the trait. The numbers of QTLs identified in these studies range from 1 to 268, using on average 227 lines and marker densities of about 10 cM (Table�2). Given these population sizes and root trait heritabilities of ≤50%, at best each would only have the power to detect half the real number of QTLs. As a consequence, the effect size of each QTL is likely to be overestimated, resulting in QTLs that cannot easily be fine-mapped without enormous effort. These are major impediments to identification of genes underlying root quantitative traits in maize.

Meta-QTL analyses have attempted to leverage the many available maize root QTL studies to home in on key loci that are in common across populations and environments (Tuberosa et�al. 2003, Hund et�al. 2011). Hund et�al. (2011) performed a meta-QTL analysis on traits relating to root length in maize from 15 QTL studies that used eight bi-parental mapping populations (15 inbreds) and one association mapping population (74 inbreds). A total of 161 single QTLs from the different studies were condensed into 24 meta-QTLs, with only 16 individual QTLs remaining. Root length traits were grouped based on axile and lateral root type and branching order, and available yield QTLs were overlaid. Seminal root meta-QTLs co-localized with yield QTLs more than any other trait. Six meta-QTLs in bins 1.07, 2.04, 2.08, 3.06, 6.05 and 7.04 were suggested as good candidates for further research and gene identification due to the number of single QTLs combined from different mapping populations and the number of different traits co-localized. For example, a meta-QTL in bin 2.04, located 15 cM from Root-ABA1 (Giuliani et�al. 2005, Landi et�al. 2007), had 10 co-localized QTLs from three populations for length and number of seminal roots, root capacitance, lateral root number and elongation rate, and root pulling force. Notably, the meta-analysis included studies from different developmental times (first/second leaf stage, silking and physiological maturity), in different environments (paper roll, hydroponics, greenhouse and field) and using different measurement techniques (manual measurements, root pulling force, root capacitance and root volume). These sources of variability are likely to limit the genetic signal that could be discerned from the meta-analysis. While comparative approaches have helped refine some of the most promising regions, they have not delivered on gene identification thus far.

When leveraging diverse germplasm for root genetic analysis, controlling for phenology, the timing of developmental events, is a key challenge, yet often ignored. The highest heritability of root crown traits and the greatest number of QTLs were observed after a longer period of growth (at silking), compared with the six leaf stage when phenological differences are not as great (Cai et�al. 2012). Both genotype and environment can lead to differences in the rates of leaf emergence and flowering times, which uncouples calendar time from developmental time in a relative sense. For example, since crown root whorl number is tied directly to leaf formation, a plant with more leaves is likely to have more crown roots if evaluated at the same time. It is then perhaps not surprising that when comparing root traits from tropical teosintes vs. the temperate maize inbred line ‘W22’, many of the QTLs controlling crown root number coincided with flowering time genes (Zhang et�al. 2018). The key flowering time locus *vgt1* is linked to a major seminal root number QTL on chromosome 8 (Salvi et�al. 2016), suggesting that phenological effects may control seminal root number. While these results could indicate that flowering time directly regulates root traits, especially in terms of size (lengths, surface areas, volumes and biomass) and numbers (of axile and primary branches), controlling for flowering time, either experimentally or statistically, could reveal important traits that scale proportionally or allometrically. Trait values may be higher in magnitude for larger root systems, but relatively greater for smaller root systems if phenology is included in the calculation. Given that ‘bigger’ is not always ‘better,’ especially in terms of crop cultivation at high plant density (Duvick 2005, Hammer et al. 2009, York et�al. 2015), and trade-offs in root vs. shoot resource allocation (Lynch et�al. 2005, Saengwilai et�al. 2014), it will be important to learn the extent to which there is genetic variation for root allometry. Both the size and relative values of traits should be considered when studying root architecture, especially in diverse germplasm from different latitudes.

Allometric relationships are also important when considering root phenotypic plasticity to the environment. Growth simulations of maize roots computed different optimal lateral root densities in response to varying nitrogen and phosphorus availability, which were corroborated by analysis of excavated root crowns (Postma et�al. 2014). Still, the genetic basis of plasticity remains notably elusive despite the fact that it has been exploited for many investigations of stress tolerance and genotype�environment (G�E) interactions (Trachsel et�al. 2013, Gao and Lynch 2016). In more cases than not, environmental and G�E factors interfere with our ability to understand root genetics. For example, when Li et�al. (2015) repeated their seedling hydroponics experiment three times and mapped QTLs for each repeated experiment, they found only 21 of 114 QTLs in common across repeated experiments. In a much simpler set of controlled environment experiments, Spalding and colleagues showed a profound effect of external environment, developmental time and seed size/composition on Arabidopsis seedling root gravitropism dynamics and their quantitative genetic basis (Durham Brooks et�al. 2010, Moore et�al. 2013). The temporal and subtle environmental dependencies of QTLs identified in these works are striking results that provide context to the current inability to replicate, co-align and move beyond initial root QTL results in maize. Clearly, future work will need to focus on capturing the dynamism of root growth (Kwak et�al. 2014) and environmental interactions, as a function of both real and developmental time.

## Acquiring Superior Phenotypes with Higher Throughput for the Investigation of the Genetic Control of Maize Root Architecture

The acquisition of root phenotype data is becoming cheaper, faster and better, largely due to rapid advances in digital imaging and automated analysis (Galkovskyi et�al. 2012, Pierret et�al. 2013, Pace et�al. 2014, Das et�al. 2015, Rell�n-�lvarez et�al. 2015, Symonova et�al. 2015). These exciting technological innovations have been reviewed extensively (Fiorani and Schurr 2013, Paez-Garcia et�al. 2015, Topp et�al. 2016, Morris et�al. 2017), but how can we best exploit this richness of information to understand the genetics of root architecture? The first maize root QTL study was limited to seminal and nodal root number, as well as root pulling force of plants grown in a soil glasshouse (Lebreton et�al. 1995). The first field study of maize root QTLs focused on nodal root numbers, angles and estimated mean diameters of excavated samples (Guingo et�al. 1998). While these measurements are intuitive and (relatively) easily quantified, they are only small samples of an entire maize root system, which can only sufficiently be described considering three dimensions and time. More traits are measured now than ever before; whereas only 2.7 traits per study were averaged prior to 2011, 8.3 traits were averaged since (Table�2), (Hund et�al. 2011). A parallel can be drawn in this progression with the effects that high-throughput genotyping and other ‘omics’ technologies had on our understanding of gene function. Many pathways previously defined as linear by classical mutational analysis are now considered as part of large and complex gene/protein networks governed at a genome-wide scale by systems-level rules (Westerhoff and Palsson 2004). Given that the phenome is vastly more complex than the genome (Houle 2010, Chitwood and Topp 2015), we can now move beyond only historically driven, a priori thinking about what traits are important to measure, and let the wealth of new data define the trait through statistical modeling in our quest to unravel genotype to phenotype relationships in roots (Bodner et�al. 2013, Topp et�al. 2016).

Multivariate statistics, applied mathematics and machine learning (ML) approaches can be particularly powerful when applied to root architecture analysis because they can extract the key relationships among multivariate and multidimensional data (Balduzzi et�al. 2017). There is currently no viable way to capture the 3D/4D shape of a maize root as it grows in the field. Therefore, important trade-offs must be made: minirhizotrons can be used to measure temporal dynamics, but only capture a small fraction of the root system and the topology is lost. Roots can be excavated to varying extents manually or mechanically, but the process is destructive and typically only coarse measurements are taken. Entire growing root systems can be imaged at high resolution using X-ray computed tomography (XRT) or other methods, but these approaches are constrained by the size and resolution of the imaging system, which limits plant and pot sizes as well as growth media. The spectra of phenotype vary in their dimensionality, units, scale, throughput, accuracy and precision, and thus require capable analysis frameworks.

For example, principal component analysis (PCA) and linear discriminant analysis (LDA) are effective statistical methods that can be used to reduce the dimensional space of data to reveal the underlying axes of variation. In Fig.�1 we show an example of how LDA can discriminate the shapes of field-excavated root crowns among three maize inbred lines. The upper left panel shows 2D photographs of each genotype, which were the inputs to the open-access Digital Imaging of Root Traits (DIRT; http://dirt.iplantcollaborative.org/ (July 29, 2018, date last accessed)) software. DIRT calculates dozens of features that describe shape variations in these root images. We applied PCA to reduce the number of traits, and took the first three PCs, which explained >90% of the variation, for LDA. Despite obvious differences in their root structures, LD1 and LD2 do not clearly group each genotype. DIRT has enabled at least an order of magnitude increase in the number of field-grown roots that can be evaluated, along with improved accuracy and precision (Bucksch et�al. 2014, Das et�al. 2015); however, information is lost by representing a complex 3D object into two dimensions. The lower left panel of Fig.�1 shows a 3D reconstruction generated from scanning the same root crowns via XRT. We measured the shapes in 3D using the RSA Gia pipeline (Topp et�al. 2013) and conducted a PCA and LDA. In this analysis, the first two linear discriminants clearly discriminate all three genotypes from one another. This result highlights how multivariate analysis coupled with enhanced phenotypes can improve our ability to make genotype to phenotype associations.

**Fig. 1:**
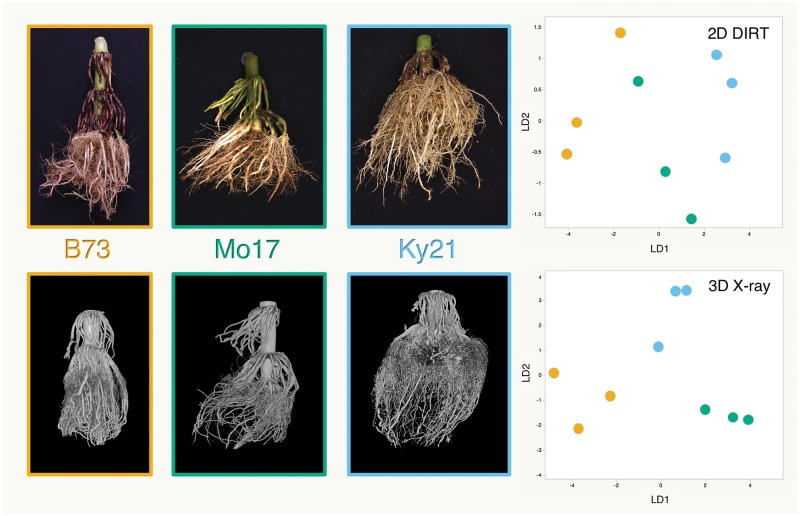
Comparison of 2D images (top) and 3D X-ray computed tomography (XRT) scans (bottom) of maize root crowns excavated from the field at tasseling. Differences between ‘B73,’ ‘Mo17’ and ‘Ky21’ from the images are visually apparent, showing the narrower, denser habit of ‘B73’ and ‘Ky21’ compared with ‘Mo17’; however, LDA of 2D DIRT traits does not entirely discriminate these genotypes (top right). Additional information gathered from 3D XRT scans and 3D GiaRoot traits group these genotypes in LDA (bottom right). These data are a subset of three excavated root crowns from three maize inbred lines collected at tasseling from a larger experiment conducted at our field site at University of Missouri Genetics Farm, Columbia, MO.

While multivariate approaches are powerful to discern subtle or pleiotropic phenotypes, their biological interpretation is sometimes called into question because the trait space is no longer easily described along a univariate axis. Instead, multivariate models often provide a new descriptor without an intuitive connection to more common traits (Topp et�al. 2013, Kenobi et�al. 2017). However, we argue that data-defined trait descriptors will play a key role in finally realizing the potential of root imaging technologies to identify genes. A 3D QTL analysis of seedling root systems in rice leveraged multivariate analysis of variance (MANOVA) to identify several large effect QTLs that were not identified by the univariate traits alone, and explained more phenotypic variation in the data (Topp et�al. 2013). Composite traits were computed from the relative contributions of each univariate trait to the multivariate, and used to verify the MANOVA-based QTLs. Thus, the very concept of genetic architecture for a complex trait such as root shape is strongly influenced by how we quantify it in the trait space. The impacts of ML on plant science will also be felt in this area. Classification-focused ML methods such as support vector machines and logistic regression have been used recently as multivariate discriminators for root phenotypes (Iyer-Pascuzzi et�al. 2010, Zurek et�al. 2015), and deep learning approaches such as convolutional neural networks are poised to learn salient features of the data that can lead to automated quantification of statistical descriptors (Pound et�al. 2017).

Multivariate methods are a powerful complement to univariate traits, but are still limited to input features that were developed a priori to capture specific aspects of root shape. A much more general statistical approach was used to quantify shoot architectures by their spatial density functions, revealing fundamental similarities in 3D Gaussian density functions across several species and stages of development that were otherwise hidden (Conn et�al. 2017a). The work followed on an evaluation of architectural trade-offs between biomass investment and resource distribution in the same samples using graph theory (Conn et�al. 2017b), which also seems fitting for root architecture studies. Topological data analysis (TDA) methods are another way to provide more comprehensive quantifications of whole plant and root shapes without pre-supposing any specific trait (Li et�al. 2017, Delory et al. 2018). A study evaluating the genetic determinants of tomato root architecture showed that a TDA approach known as persistent homology can be used for QTL analysis. Persistent homology captured nearly all of the loci that the most heritable DIRT traits did, plus nearly two dozen additional QTLs (Li et�al. 2018). These studies demonstrate that the traditional univariate trait methods of quantifying root architecture are simply not adequate to describe all of the phenotypic variation, and therefore have probably limited our ability to discern the underlying genetic relationships in maize and many other species.

## Conclusions and Outlooks

Only a small handful of genes are currently known to control maize root development, and none has been shown specifically to influence quantitative traits useful for breeding or understanding plant�environment interactions. While the genotype to phenotype gap is generally a major challenge of current biological research, it is especially wide for root biology because of limitations in phenotyping tools and analysis approaches. Nonetheless, the pace of advances has been increasing so that now higher quality, higher throughput studies can be conducted in many laboratories throughout the world, and excellent open-source platforms for quantifying, describing and modeling root phenotypes are in place (Lobet et�al. 2013, Lobet et�al. 2015, Postma et�al. 2017, Delory et�al. 2018, Schnepf et�al. 2018).

The re-evaluation of maize mutant phenotypes with current tools may be particularly informative to understand how known genes may impact quantitative aspects of root architecture, and to identify the genetic polymorphisms underlying mutants that have not yet been cloned (Table�1). High-throughput phenotyping methods coupled to the current wealth of genotype information across hundreds of maize accessions (Gore et�al. 2009, Hirsch et�al. 2014, Bukowski et�al. 2018) can also be used to scan for common polymorphisms in these genes that may modify their phenotypic expression in more subtle ways than could be previously measured. Similarly, the evaluation of known mutants in many different genetic backgrounds could reveal modifying genes that will help connect root development and architecture at a molecular systems level. It is likely to be this quantitative variation that allows us to advance our understanding of the genetic basis of complex root phenotypes. Dynamic imaging of root growth and the function-valued phenotypes derived from these data will be a key part of increasing the information content of the phenotypes we collect, which is being enabled in 3D and increasingly environmentally realistic scenarios (Fig.�1) (Clark et�al. 2011, Metzner et�al. 2015, Symonova et�al. 2015, Ahmed et�al. 2016, van Dusschoten et�al. 2016, Morris et�al. 2017). However, these tools are still nascent, not widespread, and thus only on the cusp of realizing their potential. It will be important to continue to establish sound and transparent methods for data acquisition and post-processing in order to lower the ‘barriers to entry’ for the many root biologists who wish to incorporate advanced phenotyping into their studies.

Given the crop improvement potential for root traits, a major emphasis will continue to be field studies. Belowground phenotyping capacity will always be less than aboveground (Pauli et�al. 2016), but it is now possible to harvest and evaluate hundreds or thousands of superficial root crown samples for root architecture using computer vision (Fig.�1; Table�2) (Bucksch et�al. 2014, Das et�al. 2015). Minirhizotron and soil coring data provide additional (but sparse) information of roots at depth that can be combined with root crown excavations in high throughput to provide a more complete picture of the true root structure. Such integrated phenotyping approaches could move beyond simple trait comparisons, and directly evaluate QTLs in common from complementary data types, which will be more robust than using only one approach. All things considered, it seems only a matter of time until the combined efforts of laboratory and field work, bolstered by technological and computational advances, will begin to yield a more thorough understanding of the genetics controlling the hidden half of maize.

## Funding

This material is based upon work supported by the National Science Foundation [award numbers: IIA-1355406 and IOS-1638507 to C.N.T.].

## Disclosures

The authors have no conflicts of interest to declare.

## Abbreviations

DIRT: Digital Imaging of Root Traits
DRO1: Deeper Rooting 1
G�E: genotype�environment
GWAS: genome-wide association study
LDA: linear discriminant analysis
Lrp1: Lateral root primordia 1
lrt1: lateral rootless 1
MANOVA: multivariate analysis of variance
ML: machine learning
PCA: principal component analysis
PSTOL1: Phosphorus-Starvation Tolerance 1
QTL: quantitative trait locus
rth: roothairless
RSA: root system architecture
rtcl: rtcs-like
rtcs: rootless concerning seminal and crown roots
Rul1: Rum1-like
rum1: rootless with undetectable meristem 1
slr: short lateral roots
TDA: topological data analysis
XRT: X-ray computed tomography

## References

[pcy141-B1] Abdel-Ghani A.H., Kumar B., Pace J., Jansen C., Gonzalez-Portilla P.J., Reyes-Matamoros J., et al (2015) Association analysis of genes involved in maize (*Zea mays* L.) root development with seedling and agronomic traits under contrasting nitrogen levels. Plant Mol. Biol. 88: 133–147. 25840559 10.1007/s11103-015-0314-1

[pcy141-B2] Ahmed S., Klassen T.N., Keyes S., Daly M., Jones D.L., Mavrogordato M., et al (2016) Imaging the interaction of roots and phosphate fertiliser granules using 4D X-ray tomography. Plant Soil 401: 125–134.

[pcy141-B3] Andorf C.M., Cannon E.K., Portwood J.L. 2nd, Gardiner J.M., Harper L.C., Schaeffer M.L., et al (2016) MaizeGDB update: new tools, data and interface for the maize model organism database. Nucleic Acids Res. 44: D1195–D1201. 26432828 10.1093/nar/gkv1007 PMC4702771

[pcy141-B4] Balduzzi M., Binder B.M., Bucksch A., Chang C., Hong L., Iyer-Pascuzzi A.S., et al (2017) Reshaping plant biology: qualitative and quantitative descriptors for plant morphology. Front Plant Sci. 8: 117. 28217137 10.3389/fpls.2017.00117 PMC5289971

[pcy141-B5] Beavis W.D. (1994) The power and deceit of QTL experiments: lessons from comparative QTL studies *In* Proceedings of the Forty-Ninth Annual Corn and Sorghum Industry Research Conference. pp. 250–266. ASTA, Washington.

[pcy141-B6] Bodner G., Leitner D., Nakhforoosh A., Sobotik M., Moder K., Kaul H.-P. (2013) A statistical approach to root system classification. Front. Plant Sci. 4: 292. 23914200 10.3389/fpls.2013.00292 PMC3729997

[pcy141-B7] Bucksch A., Burridge J., York L.M., Das A., Nord E., Weitz J.S., et al (2014) Image-based high-throughput field phenotyping of crop roots. Plant Physiol. 166: 470–486. 25187526 10.1104/pp.114.243519 PMC4213080

[pcy141-B8] Bukowski R., Guo X., Lu Y., Zou C., He B., Rong Z., et al (2018) Construction of the third generation *Zea mays* haplotype map. Gigascience 7: 1–12. 10.1093/gigascience/gix134 PMC5890452 29300887

[pcy141-B9] Burton A.L., Brown K.M., Lynch J.P. (2013) Phenotypic diversity of root anatomical and architectural traits in Zea species. Crop Sci. 53: 1042–1055.

[pcy141-B10] Burton A.L., Johnson J., Foerster J., Hanlon M.T., Kaeppler S.M., Lynch J.P., et al (2015) QTL mapping and phenotypic variation of root anatomical traits in maize (*Zea mays* L.). Theor. Appl. Genet. 128: 93–106. 25326723 10.1007/s00122-014-2414-8

[pcy141-B11] Burton A.L., Johnson J.M., Foerster J.M., Hirsch C.N., Buell C.R., Hanlon M.T., et al (2014) QTL mapping and phenotypic variation for root architectural traits in maize (*Zea mays* L.). Theor. Appl. Genet. 127: 2293–2311. 25230896 10.1007/s00122-014-2353-4

[pcy141-B12] Cai H., Chen F., Mi G., Zhang F., Maurer H.P., Liu W., et al (2012) Mapping QTLs for root system architecture of maize (*Zea mays* L.) in the field at different developmental stages. Theor. Appl. Genet. 125: 1313–1324. 22718302 10.1007/s00122-012-1915-6

[pcy141-B13] Chitwood D.H., Topp C.N. (2015) Revealing plant cryptotypes: defining meaningful phenotypes among infinite traits. Curr. Opin. Plant Biol. 24: 54–60. 25658908 10.1016/j.pbi.2015.01.009

[pcy141-B14] Clark R.T., MacCurdy R.B., Jung J.K., Shaff J.E., McCouch S.R., Aneshansley D.J., et al (2011) Three-dimensional root phenotyping with a novel imaging and software platform. Plant Physiol. 156: 455–465. 21454799 10.1104/pp.110.169102 PMC3177249

[pcy141-B15] Conn A., Pedmale U.V., Chory J., Navlakha S. (2017b) High-resolution laser scanning reveals plant architectures that reflect universal network design principles. Cell Syst. 5: 53–62. 28750198 10.1016/j.cels.2017.06.017

[pcy141-B16] Conn A., Pedmale U.V., Chory J., Stevens C.F., Navlakha S. (2017a) A statistical description of plant shoot architecture. Curr. Biol. 27: 2078–2088. 28690115 10.1016/j.cub.2017.06.009 PMC6130893

[pcy141-B17] Das A., Schneider H., Burridge J., Ascanio A.K.M., Wojciechowski T., Topp C.N., et al (2015) Digital imaging of root traits (DIRT): a high-throughput computing and collaboration platform for field-based root phenomics. Plant Methods 11: 51. 26535051 10.1186/s13007-015-0093-3 PMC4630929

[pcy141-B18] de Dorlodot S., Forster B., Pag�s L., Price A., Tuberosa R., Draye X. (2007) Root system architecture: opportunities and constraints for genetic improvement of crops. Trends Plant Sci. 12: 474–481. 17822944 10.1016/j.tplants.2007.08.012

[pcy141-B19] Delory B.M., Li M., Topp C.N., Lobet G. (2018) archiDART v3.0: a new data analysis pipeline allowing the topological analysis of plant root systems. F1000Res. 7: 22. 29636899 10.12688/f1000research.13541.1 PMC5871803

[pcy141-B20] Durham Brooks T.L., Miller N.D., Spalding E.P. (2010) Plasticity of Arabidopsis root gravitropism throughout a multidimensional condition space quantified by automated image analysis. Plant Physiol. 152: 206–216. 19923240 10.1104/pp.109.145292 PMC2799357

[pcy141-B21] Duvick D.N. (2005) The contribution of breeding to yield advances in maize (*Zea mays* L.). Adv. Agron. 86: 83–145.

[pcy141-B22] Feix G., Hochholdinger F., Park W.J. (2002) Maize root system and genetic analysis of its formation *In* Plant Roots: The Hidden Half, 3rd edn Edited by Waisel Y., Eshel A., Beeckman T., Kafkafi U. pp. 239–248. Marcel Dekker, New York.

[pcy141-B23] Feldman L. (1994) The maize root *In* The Maize Handbook. Edited by Greeling M., Walbot V. pp. 29–37. Springer, New York.

[pcy141-B24] Fiorani F., Schurr U. (2013) Future scenarios for plant phenotyping. Annu. Rev. Plant Biol. 64: 267–291. 23451789 10.1146/annurev-arplant-050312-120137

[pcy141-B25] Fitter A.H. (1987) An architectural approach to the comparative ecology of plant root systems. New Phytol. 106: 61–77.

[pcy141-B26] Galkovskyi T., Mileyko Y., Bucksch A., Moore B., Symonova O., Price C.A., et al (2012) GiA Roots: software for the high throughput analysis of plant root system architecture. BMC Plant Biol. 12: 116. 22834569 10.1186/1471-2229-12-116 PMC3444351

[pcy141-B27] Gamuyao R., Chin J.H., Pariasca-Tanaka J., Pesaresi P., Catausan S., Dalid C., et al (2012) The protein kinase Pstol1 from traditional rice confers tolerance of phosphorus deficiency. Nature 488: 535–539. 22914168 10.1038/nature11346

[pcy141-B28] Gao Y., Lynch J.P. (2016) Reduced crown root number improves water acquisition under water deficit stress in maize (*Zea mays* L.). J. Exp. Bot. 67: 4545–4557. 27401910 10.1093/jxb/erw243 PMC4973737

[pcy141-B29] Gifford M.L., Banta J.A., Katari M.S., Hulsmans J., Chen L., Ristova D., et al (2013) Plasticity regulators modulate specific root traits in discrete nitrogen environments. PLoS Genet. 9: e1003760. 24039603 10.1371/journal.pgen.1003760 PMC3764102

[pcy141-B30] Giuliani S., Sanguineti M.C., Tuberosa R., Bellotti M., Salvi S., Landi P. (2005) Root-ABA1, a major constitutive QTL, affects maize root architecture and leaf ABA concentration at different water regimes. J. Exp. Bot. 56: 3061–3070. 16246858 10.1093/jxb/eri303

[pcy141-B31] Gore M.A., Chia J.-M., Elshire R.J., Sun Q., Ersoz E.S., Hurwitz B.L., et al (2009) A first-generation haplotype map of maize. Science 326: 1115–1117. 19965431 10.1126/science.1177837

[pcy141-B32] Gu D., Mei X., Yu T., Sun N., Xu D., Liu C., et al (2017) QTL identification for brace-root traits of maize in different generations and environments. Crop Sci. 57: 13–21.

[pcy141-B33] Guingo E., H�bert Y., Charcosset A. (1998) Genetic analysis of root traits in maize. Agronomie 18: 225–235.

[pcy141-B151] Hammer G.L., Dong Z., McLean G., Doherty A., Messina C., Schussler J., et al (2009) Can changes in canopy and/or root system architecture explain historical maize yield trends in the US corn belt? Crop Sci. 49: 299–312.

[pcy141-B34] Hirsch C.N., Foerster J.M., Johnson J.M., Sekhon R.S., Muttoni G., Vaillancourt B., et al (2014) Insights into the maize pan-genome and pan-transcriptome. Plant Cell 26: 121–135. 24488960 10.1105/tpc.113.119982 PMC3963563

[pcy141-B35] Hirsch C.N., Hirsch C.D., Brohammer A.B., Bowman M.J., Soifer I., Barad O., et al (2016) Draft assembly of elite inbred line PH207 provides insights into genomic and transcriptome diversity in maize. Plant Cell 28: 2700–2714. 27803309 10.1105/tpc.16.00353 PMC5155341

[pcy141-B36] Hochholdinger F. (2009) The maize root system: morphology, anatomy, and genetics *In* Handbook of Maize: Its Biology. Edited by Bennetzen J.L., Hake S.C. pp. 145–160. Springer, New York.

[pcy141-B37] Hochholdinger F., Feix G. (1998) Early post-embryonic root formation is specifically affected in the maize mutant lrt1. Plant J. 16: 247–255. 22507137 10.1046/j.1365-313x.1998.00280.x

[pcy141-B38] Hochholdinger F., Park W.J., Feix G.H. (2001) Cooperative action of SLR1 and SLR2 is required for lateral root-specific cell elongation in maize. Plant Physiol. 125: 1529–1539. 11244131 10.1104/pp.125.3.1529 PMC65630

[pcy141-B39] Hochholdinger F., Tuberosa R. (2009) Genetic and genomic dissection of maize root development and architecture. Curr. Opin. Plant Biol. 12: 172–177. 19157956 10.1016/j.pbi.2008.12.002

[pcy141-B40] Hochholdinger F., Wen T.-J., Zimmermann R., Chimot-Marolle P., da Costa e Silva O., Bruce W., et al (2008) The maize (*Zea mays* L.) roothairless3 gene encodes a putative GPI-anchored, monocot-specific, COBRA-like protein that significantly affects grain yield. Plant J. 54: 888–898. 18298667 10.1111/j.1365-313X.2008.03459.x PMC2440564

[pcy141-B41] Hochholdinger F., Woll K., Sauer M., Feix G. (2005) Functional genomic tools in support of the genetic analysis of root development in maize (*Zea mays* L. ). Maydica 50: 437–442.

[pcy141-B42] Hochholdinger F., Yu P., Marcon C. (2018) Genetic control of root system development in maize. Trends Plant Sci. 23: 79–88. 29170008 10.1016/j.tplants.2017.10.004

[pcy141-B43] Houle D. (2010) Colloquium papers: numbering the hairs on our heads: the shared challenge and promise of phenomics. Proc. Natl. Acad. Sci. USA 107: 1793–1799. 19858477 10.1073/pnas.0906195106 PMC2868290

[pcy141-B44] Hufnagel B., de Sousa S.M., Assis L., Guimaraes C.T., Leiser W., Azevedo G.C., et al (2014) Duplicate and conquer: multiple homologs of PHOSPHORUS-STARVATION TOLERANCE1 enhance phosphorus acquisition and sorghum performance on low-phosphorus soils. Plant Physiol. 166: 659–677. 25189534 10.1104/pp.114.243949 PMC4213096

[pcy141-B45] Hund A., Reimer R., Messmer R. (2011) A consensus map of QTLs controlling the root length of maize. Plant Soil 344: 143–158.

[pcy141-B46] Ilic K., Kellogg E.A., Jaiswal P., Zapata F., Stevens P.F., Vincent L.P., et al (2007) The plant structure ontology, a unified vocabulary of anatomy and morphology of a flowering plant. Plant Physiol. 143: 587–599. 17142475 10.1104/pp.106.092825 PMC1803752

[pcy141-B47] Iyer-Pascuzzi A.S., Symonova O., Mileyko Y., Hao Y., Belcher H., Harer J., et al (2010) Imaging and analysis platform for automatic phenotyping and trait ranking of plant root systems. Plant Physiol. 152: 1148–1157. 20107024 10.1104/pp.109.150748 PMC2832248

[pcy141-B48] Jenkins M.T. (1930) Heritable characters of maize: XXXIV—rootless. J. Hered. 21: 79–80.

[pcy141-B49] Jiao Y., Peluso P., Shi J., Liang T., Stitzer M.C., Wang B., et al (2017) Improved maize reference genome with single-molecule technologies. Nature 546: 524–527. 28605751 10.1038/nature22971 PMC7052699

[pcy141-B50] Kenobi K., Atkinson J.A., Wells D.M., Gaju O., De Silva J.G., Foulkes M.J., et al (2017) Linear discriminant analysis reveals differences in root architecture in wheat seedlings related to nitrogen uptake efficiency. J. Exp. Bot. 68: 4969–4981. 29048563 10.1093/jxb/erx300 PMC5853436

[pcy141-B51] Kiesselbach T.A. (1949) The Structure and Reproduction of Corn Agricultural Experimental Station Research Bulletin No. 161. University of Nebraska College of Agriculture

[pcy141-B52] Ku L.X., Sun Z.H., Wang C.L., Zhang J., Zhao R.F., Liu H.Y., et al (2012) QTL mapping and epistasis analysis of brace root traits in maize. Mol. Breed. 30: 697–708.

[pcy141-B53] Kwak I.-Y., Moore C.R., Spalding E.P., Broman K.W. (2014) A simple regression-based method to map quantitative trait loci underlying function-valued phenotypes. Genetics 197: 1409–1416. 24931408 10.1534/genetics.114.166306 PMC4125409

[pcy141-B54] Landi P., Sanguineti M.C., Liu C., Li Y., Wang T.Y., Giuliani S., et al (2007) Root-ABA1 QTL affects root lodging, grain yield, and other agronomic traits in maize grown under well-watered and water-stressed conditions. J. Exp. Bot. 58: 319–326. 17050640 10.1093/jxb/erl161

[pcy141-B55] Lebreton C., Lazić-Jančić V., Steed A., Pekić S., Quarrie S.A. (1995) Identification of QTL for drought responses in maize and their use in testing causal relationships between traits. J. Exp. Bot. 46: 853–865.

[pcy141-B56] Li L., Hey S., Liu S., Liu Q., McNinch C., Hu H.-C., et al (2016) Characterization of maize roothairless6 which encodes a D-type cellulose synthase and controls the switch from bulge formation to tip growth. Sci. Rep. 6: 34395. 27708345 10.1038/srep34395 PMC5052636

[pcy141-B57] Li M., Duncan K., Topp C.N., Chitwood D.H. (2017) Persistent homology and the branching topologies of plants. Amer. J. Bot. 104: 349–353. 28341629 10.3732/ajb.1700046

[pcy141-B58] Li M., Frank M., Coneva V., Mio W., Chitwood D.H., Topp C.N. (2018) The persistent homology mathematical framework provides enhanced genotype-to-phenotype associations for plant morphology. Plant Physiol. bioRxiv. doi:10.1101/104141. 10.1104/pp.18.00104 PMC6084663 29871979

[pcy141-B59] Li P., Chen F., Cai H., Liu J., Pan Q., Liu Z., et al (2015) A genetic relationship between nitrogen use efficiency and seedling root traits in maize as revealed by QTL analysis. J. Exp. Bot. 66: 3175–3188. 25873660 10.1093/jxb/erv127 PMC4449538

[pcy141-B60] Liu J., Cai H., Chu Q., Chen X., Chen F., Yuan L., et al (2011) Genetic analysis of vertical root pulling resistance (VRPR) in maize using two genetic populations. Mol. Breed. 28: 463–474.

[pcy141-B61] Liu Z., Gao K., Shan S., Gu R., Wang Z., Craft E.J., et al (2017) Comparative analysis of root traits and the associated QTLs for maize seedlings grown in paper roll, hydroponics and vermiculite culture system. Front. Plant Sci. 8: 436. 28424719 10.3389/fpls.2017.00436 PMC5371678

[pcy141-B62] Lobet G., Draye X., P�rilleux C. (2013) An online database for plant image analysis software tools. Plant Methods 9: 38. 24107223 10.1186/1746-4811-9-38 PMC3853381

[pcy141-B63] Lobet G., Pound M.P., Diener J., Pradal C., Draye X., Godin C., et al (2015) Root system markup language: toward a unified root architecture description language. Plant Physiol. 167: 617–627. 25614065 10.1104/pp.114.253625 PMC4348768

[pcy141-B64] Lynch J. (1995) Root architecture and plant productivity. Plant Physiol. 109: 7–13. 12228579 10.1104/pp.109.1.7 PMC157559

[pcy141-B65] Lynch J.P. (2013) Steep, cheap and deep: an ideotype to optimize water and N acquisition by maize root systems. Ann. Bot. 112: 347–357. 23328767 10.1093/aob/mcs293 PMC3698384

[pcy141-B66] Lynch J.P., Chimungu J.G., Brown K.M. (2014) Root anatomical phenes associated with water acquisition from drying soil: targets for crop improvement. J. Exp. Bot. 65: 6155–6166. 24759880 10.1093/jxb/eru162

[pcy141-B67] Lynch J.P., Ho M.D., Phosphorus L. (2005) Rhizoeconomics: carbon costs of phosphorus acquisition. Plant Soil 269: 45–56.

[pcy141-B68] MacMillan K., Emrich K., Piepho H.-P., Mullins C.E., Price A.H. (2006) Assessing the importance of genotype � environment interaction for root traits in rice using a mapping population II: conventional QTL analysis. Theor. Appl. Genet. 113: 953–964. 16896715 10.1007/s00122-006-0357-4

[pcy141-B69] Mano Y., Omori F. (2008) Verification of QTL controlling root aerenchyma formation in a maize � teosinte ‘*Zea nicaraguensis*’ advanced backcross population. Breed. Sci. 58: 217–223.

[pcy141-B70] Mano Y., Omori F. (2009) High-density linkage map around the root aerenchyma locus Qaer1.06 in the backcross populations of maize Mi29 � teosinte ‘*Zea nicaraguensis*’. Breed. Sci. 59: 427–433.

[pcy141-B71] Mano Y., Omori F., Takamizo T., Kindiger B., McK Bird R., Loaisiga C.H., et al (2007) QTL mapping of root aerenchyma formation in seedlings of a maize � rare teosinte ‘*Zea nicaraguensis*’ cross. Plant Soil 295: 103–113.

[pcy141-B72] Meij�n M., Satbhai S.B., Tsuchimatsu T., Busch W. (2014) Genome-wide association study using cellular traits identifies a new regulator of root development in Arabidopsis. Nat. Genet. 46: 77–81. 24212884 10.1038/ng.2824

[pcy141-B73] Metzner R., Eggert A., van Dusschoten D., Pflugfelder D., Gerth S., Schurr U., et al (2015) Direct comparison of MRI and X-ray CT technologies for 3D imaging of root systems in soil: potential and challenges for root trait quantification. Plant Methods 11: 17. 25774207 10.1186/s13007-015-0060-z PMC4359488

[pcy141-B74] Moore C.R., Johnson L.S., Kwak I.-Y., Livny M., Broman K.W., Spalding E.P. (2013) High-throughput computer vision introduces the time axis to a quantitative trait map of a plant growth response. Genetics 195: 1077–1086. 23979570 10.1534/genetics.113.153346 PMC3813838

[pcy141-B75] Morris E.C., Griffiths M., Golebiowska A., Mairhofer S., Burr-Hersey J., Goh T., et al (2017) Shaping 3D root system architecture. Curr. Biol. 27: R919–R930. 28898665 10.1016/j.cub.2017.06.043

[pcy141-B76] Nestler J., Liu S., Wen T.-J., Paschold A., Marcon C., Tang H.M., et al (2014) Roothairless5, which functions in maize (*Zea mays* L.) root hair initiation and elongation encodes a monocot-specific NADPH oxidase. Plant J. 79: 729–740. 24902980 10.1111/tpj.12578

[pcy141-B77] Pace J., Gardner C., Romay C., Ganapathysubramanian B., L�bberstedt T. (2015) Genome-wide association analysis of seedling root development in maize (*Zea mays* L.). BMC Genomics 16: 47. 25652714 10.1186/s12864-015-1226-9 PMC4326187

[pcy141-B78] Pace J., Lee N., Naik H.S., Ganapathysubramanian B., L�bberstedt T. (2014) Analysis of maize (*Zea mays* L.) seedling roots with the high-throughput image analysis tool ARIA (Automatic Root Image Analysis). PLoS One 9: e108255. 25251072 10.1371/journal.pone.0108255 PMC4176968

[pcy141-B79] Paez-Garcia A., Motes C.M., Scheible W.-R., Chen R., Blancaflor E.B., Monteros M.J. (2015) Root traits and phenotyping strategies for plant improvement. Plants (Basel) 4: 334–355. 27135332 10.3390/plants4020334 PMC4844329

[pcy141-B80] Pauli D., Chapman S.C., Bart R., Topp C.N., Lawrence-Dill C.J., Poland J., et al (2016) The quest for understanding phenotypic variation via integrated approaches in the field environment. Plant Physiol. 172: 622–634. 27482076 10.1104/pp.16.00592 PMC5047081

[pcy141-B81] Pestsova E., Lichtblau D., Wever C., Presterl T., Bolduan T., Ouzunova M., et al (2016) QTL mapping of seedling root traits associated with nitrogen and water use efficiency in maize. Euphytica 209: 585–602.

[pcy141-B82] Pierret A., Gonkhamdee S., Jourdan C., Maeght J.-L. (2013) IJ_Rhizo: an open-source software to measure scanned images of root samples. Plant Soil 373: 531–539.

[pcy141-B83] Postma J.A., Dathe A., Lynch J.P. (2014) The optimal lateral root branching density for maize depends on nitrogen and phosphorus availability. Plant Physiol. 166: 590–602. 24850860 10.1104/pp.113.233916 PMC4213091

[pcy141-B84] Postma J.A., Kuppe C., Owen M.R., Mellor N., Griffiths M., Bennett M.J., et al (2017) OpenSimRoot: widening the scope and application of root architectural models. New Phytol. 215: 1274–1286. 28653341 10.1111/nph.14641 PMC5575537

[pcy141-B85] Pound M.P., Atkinson J.A., Townsend A.J., Wilson M.H., Griffiths M., Jackson A.S., et al (2017) Deep machine learning provides state-of-the-art performance in image-based plant phenotyping. Gigascience 6: 1–10. 10.1093/gigascience/gix083 PMC5632296 29020747

[pcy141-B86] Price A.H., Tomos A.D. (1997) Genetic dissection of root growth in rice (*Oryza sativa* L.). II: mapping quantitative trait loci using molecular markers. Theor. Appl. Genet. 95: 143–152.

[pcy141-B87] Rell�n-�lvarez R., Lobet G., Lindner H., Pradier P.-L., Sebastian J., Yee M.-C., et al (2015) GLO-Roots: an imaging platform enabling multidimensional characterization of soil-grown root systems. Elife 4. 10.7554/eLife.07597 PMC4589753 26287479

[pcy141-B88] Rosas U., Cibrian-Jaramillo A., Ristova D., Banta J.A., Gifford M.L., Fan A.H., et al (2013) Integration of responses within and across Arabidopsis natural accessions uncovers loci controlling root systems architecture. Proc. Natl. Acad. Sci. USA 110: 15133–15138. 23980140 10.1073/pnas.1305883110 PMC3773737

[pcy141-B89] Saengwilai P., Tian X., Lynch J.P. (2014) Low crown root number enhances nitrogen acquisition from low-nitrogen soils in maize. Plant Physiol. 166: 581–589. 24706553 10.1104/pp.113.232603 PMC4213090

[pcy141-B90] Salvi S., Giuliani S., Ricciolini C., Carraro N., Maccaferri M., Presterl T., et al (2016) Two major quantitative trait loci controlling the number of seminal roots in maize co-map with the root developmental genes *rtcs* and *rum1*. J. Exp. Bot. 67: 1149–1159. 26880748 10.1093/jxb/erw011 PMC4753855

[pcy141-B91] Sanchez D.L., Liu S., Ibrahim R., Blanco M., L�bberstedt T. (2018) Genome-wide association studies of doubled haploid exotic introgression lines for root system architecture traits in maize (*Zea mays* L.). Plant Sci. 268: 30–38. 29362081 10.1016/j.plantsci.2017.12.004

[pcy141-B92] Sandhu N., Raman K.A., Torres R.O., Audebert A., Dardou A., Kumar A., et al (2016) Rice root architectural plasticity traits and genetic regions for adaptability to variable cultivation and stress conditions. Plant Physiol. 171: 2562–2576. 27342311 10.1104/pp.16.00705 PMC4972292

[pcy141-B93] Sass J.E. (1977) Morphology *In* Corn and Corn Improvement. Edited by Sprague G.F., Eberhart S.A. pp. 89–110. American Society of Agronomy, Madison, WI.

[pcy141-B94] Schmidt J.E., Bowles T.M., Gaudin A.C.M. (2016) Using ancient traits to convert soil health into crop yield: impact of selection on maize root and rhizosphere function. Front. Plant Sci. 7: 373. 27066028 10.3389/fpls.2016.00373 PMC4811947

[pcy141-B95] Schnable J.C., Freeling M. (2011) Genes identified by visible mutant phenotypes show increased bias toward one of two subgenomes of maize. PLoS One 6: e17855. 21423772 10.1371/journal.pone.0017855 PMC3053395

[pcy141-B96] Schnable J.C., Springer N.M., Freeling M. (2011) Differentiation of the maize subgenomes by genome dominance and both ancient and ongoing gene loss. Proc. Natl. Acad. Sci. USA 108: 4069–4074. 21368132 10.1073/pnas.1101368108 PMC3053962

[pcy141-B97] Schnepf A., Leitner D., Landl M., Lobet G., Mai T.H., Morandage S., et al (2018) CRootBox: a structural–functional modelling framework for root systems. Ann. Bot. 121: 1033–1053. 29432520 10.1093/aob/mcx221 PMC5906965

[pcy141-B98] Symonova O., Topp C.N., Edelsbrunner H. (2015) DynamicRoots: a software platform for the reconstruction and analysis of growing plant roots. PLoS One 10: e0127657. 26030757 10.1371/journal.pone.0127657 PMC4452513

[pcy141-B99] Taramino G., Sauer M., Stauffer J.L., Multani D., Niu X., Sakai H., et al (2007) The maize (*Zea mays* L.) RTCS gene encodes a LOB domain protein that is a key regulator of embryonic seminal and post-embryonic shoot-borne root initiation. Plant J. 50: 649–659. 17425722 10.1111/j.1365-313X.2007.03075.x

[pcy141-B100] Topp C.N., Bray A.L., Ellis N.A., Liu Z. (2016) How can we harness quantitative genetic variation in crop root systems for agricultural improvement? J. Integr. Plant Biol. 58: 213–225. 26911925 10.1111/jipb.12470

[pcy141-B101] Topp C.N., Iyer-Pascuzzi A.S., Anderson J.T., Lee C.-R., Zurek P.R., Symonova O., et al (2013) 3D phenotyping and quantitative trait locus mapping identify core regions of the rice genome controlling root architecture. Proc. Natl. Acad. Sci. USA 110: E1695–E1704. 23580618 10.1073/pnas.1304354110 PMC3645568

[pcy141-B102] Trachsel S., Kaeppler S.M., Brown K.M., Lynch J.P. (2013) Maize root growth angles become steeper under low N conditions. Field Crops Res. 140: 18–31.

[pcy141-B103] Tuberosa R., Salvi S., Sanguineti M.C., Maccaferri M., Giuliani S., Landi P. (2003) Searching for quantitative trait loci controlling root traits in maize: a critical appraisal. Plant Soil 255: 35–54.

[pcy141-B104] Uga Y., Sugimoto K., Ogawa S., Rane J., Ishitani M., Hara N., et al (2013) Control of root system architecture by DEEPER ROOTING 1 increases rice yield under drought conditions. Nat. Genet. 45: 1097–1102. 23913002 10.1038/ng.2725

[pcy141-B105] van Dusschoten D., Metzner R., Kochs J., Postma J.A., Pflugfelder D., B�hler J., et al (2016) Quantitative 3D analysis of plant roots growing in soil using magnetic resonance imaging. Plant Physiol. 170: 1176–1188. 26729797 10.1104/pp.15.01388 PMC4775118

[pcy141-B106] von Behrens I., Komatsu M., Zhang Y., Berendzen K.W., Niu X., Sakai H., et al (2011) Rootless with undetectable meristem 1 encodes a monocot-specific AUX/IAA protein that controls embryonic seminal and post-embryonic lateral root initiation in maize: AUX/IAA regulation of maize root development. Plant J. 66: 341–353. 21219511 10.1111/j.1365-313X.2011.04495.x

[pcy141-B107] Watanabe K., Takahashi H., Sato S., Nishiuchi S., Omori F., Malik A.I., et al (2017) A major locus involved in the formation of the radial oxygen loss barrier in adventitious roots of teosinte *Zea nicaraguensis* is located on the short-arm of chromosome 3. Plant Cell Environ. 40: 304–316. 27762444 10.1111/pce.12849

[pcy141-B108] Wen T.-J., Hochholdinger F., Sauer M., Bruce W., Schnable P.S. (2005) The roothairless1 gene of maize encodes a homolog of sec3, which is involved in polar exocytosis. Plant Physiol. 138: 1637–1643. 15980192 10.1104/pp.105.062174 PMC1176433

[pcy141-B109] Wen T.-J., Schnable P.S. (1994) Analyses of mutants of three genes that influence root hair development in *Zea mays* (Gramineae) suggest that root hairs are dispensable. Amer. J. Bot. 81: 833–842.

[pcy141-B110] Westerhoff H.V., Palsson B.O. (2004) The evolution of molecular biology into systems biology. Nat. Biotechnol. 22: 1249–1252. 15470464 10.1038/nbt1020

[pcy141-B111] Woll K., Borsuk L.A., Stransky H., Nettleton D., Schnable P.S., Hochholdinger F. (2005) Isolation, characterization, and pericycle-specific transcriptome analyses of the novel maize lateral and seminal root initiation mutant rum1. Plant Physiol. 139: 1255–1267. 16215225 10.1104/pp.105.067330 PMC1283763

[pcy141-B112] Xu S. (2003) Theoretical basis of the Beavis effect. Genetics 165: 2259–2268. 14704201 10.1093/genetics/165.4.2259 PMC1462909

[pcy141-B113] Xu C., Tai H., Saleem M., Ludwig Y., Majer C., Berendzen K.W., et al (2015) Cooperative action of the paralogous maize lateral organ boundaries (LOB) domain proteins RTCS and RTCL in shoot-borne root formation. New Phytol. 207: 1123–1133. 25902765 10.1111/nph.13420

[pcy141-B114] York L.M., Galindo-Casta�eda T., Schussler J.R., Lynch J.P. (2015) Evolution of US maize (*Zea mays* L.) root architectural and anatomical phenes over the past 100 years corresponds to increased tolerance of nitrogen stress. J. Exp. Bot. 66: 2347–2358. 25795737 10.1093/jxb/erv074 PMC4407655

[pcy141-B115] Yu P., Gutjahr C., Li C., Hochholdinger F. (2016) Genetic control of lateral root formation in cereals. Trends Plant Sci. 21: 951–961. 27524642 10.1016/j.tplants.2016.07.011

[pcy141-B116] Zaidi P.H., Seetharam K., Krishna G., Krishnamurthy L., Gajanan S., Babu R., et al (2016) Genomic regions associated with root traits under drought stress in tropical maize (*Zea mays* L.). PLoS One 11: e0164340. 27768702 10.1371/journal.pone.0164340 PMC5074786

[pcy141-B117] Zhang Y., Marcon C., Tai H., von Behrens I., Ludwig Y., Hey S., et al (2016) Conserved and unique features of the homeologous maize Aux/IAA proteins ROOTLESS WITH UNDETECTABLE MERISTEM 1 and RUM1-like 1. J. Exp. Bot. 67: 1137–1147. 26672614 10.1093/jxb/erv519 PMC4753850

[pcy141-B118] Zhang Y., von Behrens I., Zimmermann R., Ludwig Y., Hey S., Hochholdinger F. (2015) LATERAL ROOT PRIMORDIA 1 of maize acts as a transcriptional activator in auxin signalling downstream of the Aux/IAA gene *rootless with undetectable meristem 1*. J. Exp. Bot. 66: 3855–3863. 25911745 10.1093/jxb/erv187 PMC4473986

[pcy141-B119] Zhang Z., Zhang X., Lin Z., Wang J., Xu M., Lai J., et al (2018) The genetic architecture of nodal root number in maize. Plant J. 93: 1032–1044. 29364547 10.1111/tpj.13828

[pcy141-B120] Zobel R.W., Waisel Y. (2010) A plant root system architectural taxonomy: a framework for root nomenclature. Plant Biosyst. 144: 507–512.

[pcy141-B121] Zurek P.R., Topp C.N., Benfey P.N. (2015) Quantitative trait locus mapping reveals regions of the maize genome controlling root system architecture. Plant Physiol. 167: 1487–1496. 25673779 10.1104/pp.114.251751 PMC4378147

